# Serum soluble-Fas, inflammation and anemia in acute renal failure and critical illness

**DOI:** 10.1186/cc10182

**Published:** 2011-06-22

**Authors:** MA Góes, MA Dalboni, BMR Quinto, IJ Iizuka, JC Monte, OF Pavão dos Santos, VG Pereira, M de Souza Durão, MC Batista, M Cendoroglo

**Affiliations:** 1 Federal University of São Paulo - UNIFESP, São Paulo - SP, Brazil; 2 Hospital Israelita Albert Einstein, São Paulo - SP, Brazil; 3 New England Medical Center, Tufts School of Medicine, Boston, MA, USA

## Introduction

Soluble Fas (sFas) levels are associated with anemia and erythropoietin (Epo) hyporesponsiveness in chronic kidney disease. Anemia is also a common feature in patients with acute renal failure (ARF) and in critically ill patients. Therefore, it is possible that sFas levels are also associated with anemia and increased need for serum Epo levels in ARF and critical illness in order to maintain hemoglobin (Hgb) levels.

## Objective

To investigate the relationship between serum levels of sFas, Epo, inflammatory cytokines and Hgb levels in patients with ARF and critically ill patients.

## Methods

We studied 72 critically ill patients with ARF on continuous hemodiafiltration (CVVHDF group; *n *= 53) or without ARF (non-ARF group; *n *= 19), 29 chronic hemodialysis patients (ESRD group) and 29 healthy volunteers (Healthy group). The CVVHDF dose was 30 ml/kg per hour or higher. We investigated among the four groups the relationships between Hgb and serum levels of sFas, Epo, TNFα, IL-6, IL-10 and iron status.

## Results

The CVVHDF and non-ARF groups had higher serum levels of Epo, IL-6, IL-10 and ferritin than the other groups. Hgb levels were lower in the CVVHDF group than in the other groups. Serum sFas levels were higher in uremic patients (CVVHDF and ESRD groups; *P *< 0.001). When all critically ill patients were pooled together, Hgb levels correlated negatively with serum levels of IL-6 (*r *= -0.55, *P *= 0.001), sFas (*r *= -0.40, *P *= 0.001), TNFα (r = -0.37, *P *< 0.001), iron (*r *= -0.28, *P *= 0.02), ferritin (*r *= -0.35, *P *= 0.004) and transferrin saturation (*r *= -0.30, *P *= 0.01). In multivariate analysis, after adjusting for markers of iron store and inflammation, levels of IL-6 (*P *< 0.001), sFas (*P *< 0.001) and TNFα (*P *= 0.01) correlated negatively with Hgb in critically ill patients.

## Conclusion

Our findings demonstrate that sFas is associated with anemia in ARF and critically ill patients. Serum sFas and Epo levels were higher and Hgb levels were lower in critically ill patients with ARF, suggesting that sFas may be associated with Epo hyporesponsiveness in ARF and critical illness.

